# Analysis of Protein Composition and Bioactivity of *Neoponera villosa* Venom (Hymenoptera: Formicidae)

**DOI:** 10.3390/ijms17040513

**Published:** 2016-04-21

**Authors:** Wallace Felipe Blohem Pessoa, Ludimilla Carvalho Cerqueira Silva, Leila de Oliveira Dias, Jacques Hubert Charles Delabie, Helena Costa, Carla Cristina Romano

**Affiliations:** 1 State University of Santa Cruz (UESC)—Center of Biotechnology and Genetics (CBG), Ilhéus, Bahia 45662-900, Brazil; wall.bmd@gmail.com (W.F.B.P.); ludicsilva@hotmail.com (L.C.C.S.); leila.bmd@gmail.com (L.d.O.D.); hcosta@uesc.br (H.C.); 2 Myrmecology Laboratory of the Cocoa Research Center—CEPEC, Executive Committee of the Cocoa Crop (CEPLAC), Ilhéus, Bahia 45660-000, Brazil; jacques.delabie@gmail.com

**Keywords:** ant, proteome, venomics, hemolysis, immunostimulatory proteins

## Abstract

Ants cause a series of accidents involving humans. Such accidents generate different reactions in the body, ranging from a mild irritation at the bite site to anaphylactic shock, and these reactions depend on the mechanism of action of the venom. The study of animal venom is a science known as venomics. Through venomics, the composition of the venom of several ant species has already been characterized and their biological activities described. Thus, the aim of this study was to evaluate the protein composition and biological activities (hemolytic and immunostimulatory) of the venom of *Neoponera villosa* (*N. villosa*), an ant widely distributed in South America. The protein composition was evaluated by proteomic techniques, such as two-dimensional electrophoresis. To assess the biological activity, hemolysis assay was carried out and cytokines were quantified after exposure of macrophages to the venom. The venom of *N. villosa* has a profile composed of 145 proteins, including structural and metabolic components (e.g., tubulin and ATPase), allergenic and immunomodulatory proteins (arginine kinase and heat shock proteins (HSPs)), protective proteins of venom (superoxide dismutase (SOD) and catalase) and tissue degradation proteins (hyaluronidase and phospholipase A2). The venom was able to induce hemolysis in human erythrocytes and also induced release of both pro-inflammatory cytokines, as the anti-inflammatory cytokine release by murine macrophages. These results allow better understanding of the composition and complexity of *N. villosa* venom in the human body, as well as the possible mechanisms of action after the bite.

## 1. Introduction

Accidents involving venomous animals, especially insects belonging to the order Hymenoptera, generate a variety of clinical and histopathological manifestations that range from mild irritation at the site of the sting to a severe anaphylactic reaction, often fatal [1,2].

Studies focused on venom, which examine the venom composition and mechanisms of action, have increased and, through these, several bioactive molecules have been isolated and/or identified, often of protein character [3]. We highlight studies on venoms of spiders, snakes, bees, wasps, ants, scorpions, centipedes, and frogs [4,5,6,7,8,9,10,11]. These studies have shown that the bioactivity of venom is related mainly to the release of cytokines as well as inflammatory (e.g., nitric oxide) and lipid (e.g., prostaglandins) mediators [12]. Yi *et al.* [13] showed that the ant venom from *Solenopsis invicta* acts on the nitric oxide synthase enzyme of murine macrophages, interfering in the production and release of this mediator. Similar effects were demonstrated in macrophages exposed to the venom from other animals such as scorpions, wasps, bees and spiders [14,15,16,17]. In addition, some venom presents hemolytic and/or cytotoxic activity leading to death, usually by apoptosis in human cells [16,18,19].

In the Neotropical *Neoponera* genus (Formicidae, Ponerinae), 57 ant species are described [20]. Ants of this genus possess a painful bite, level 2 on a pain scale ranging from 1 to 4 [21] that is attributed to the presence of cyclic dipeptides, as already described for the *Neoponera apicalis* venom [22]. Although the species of the Ponerinae are very close, several effects inherent to the action of the venom are reported. In insects, the venom initially causes a rapid, reversible, and dose-dependent paralysis, followed by an irreversible paralysis with subsequent permanent paralysis and death. The concentrations that lead to such manifestations vary from 38.7 to 799.2 μg/g, depending on the species of Ponerinae (which includes *Neoponera*) [23]. The main function of the venom of the genus of these ants is to capture preys and defend the colony, which is the reason why it has a paralytic effect [24].

Protein characterization and biological activity studies have become common for ant venom and, although there are some in this subject focused on the venom from other species of Ponerinae, such as *Neoponera goeldii*, *Brachyponera. chinensis*, and *Brachyponera. sennaarensis* [23,25,26], there are no other studies related to the *Neoponera villosa* venom, an ant that is found worldwide. In America, it can be found from the southwestern United States of America to southern Brazil [27], and is widely distributed throughout the Brazilian territory and is very common in the state of Bahia [28,29].

Thus, the aim of this study was to evaluate the protein composition and identify the hemolytic activity, cytotoxicity, and cytokine production induced by the *N. villosa* venom, collected in the south of the state of Bahia, Brazil.

## 2. Results

### 2.1. Doses of Protein

The proteins present in venom were quantified using the 2D Quant Kit (GE Healthcare, Little Chalfont, UK) and showed 6.23 µg/µL. By the Bradford method [30], a protein concentration of 2.79 µg/µL was found.

### 2.2. Protein Profile Obtained by SDS-PAGE

**Figure 1:**
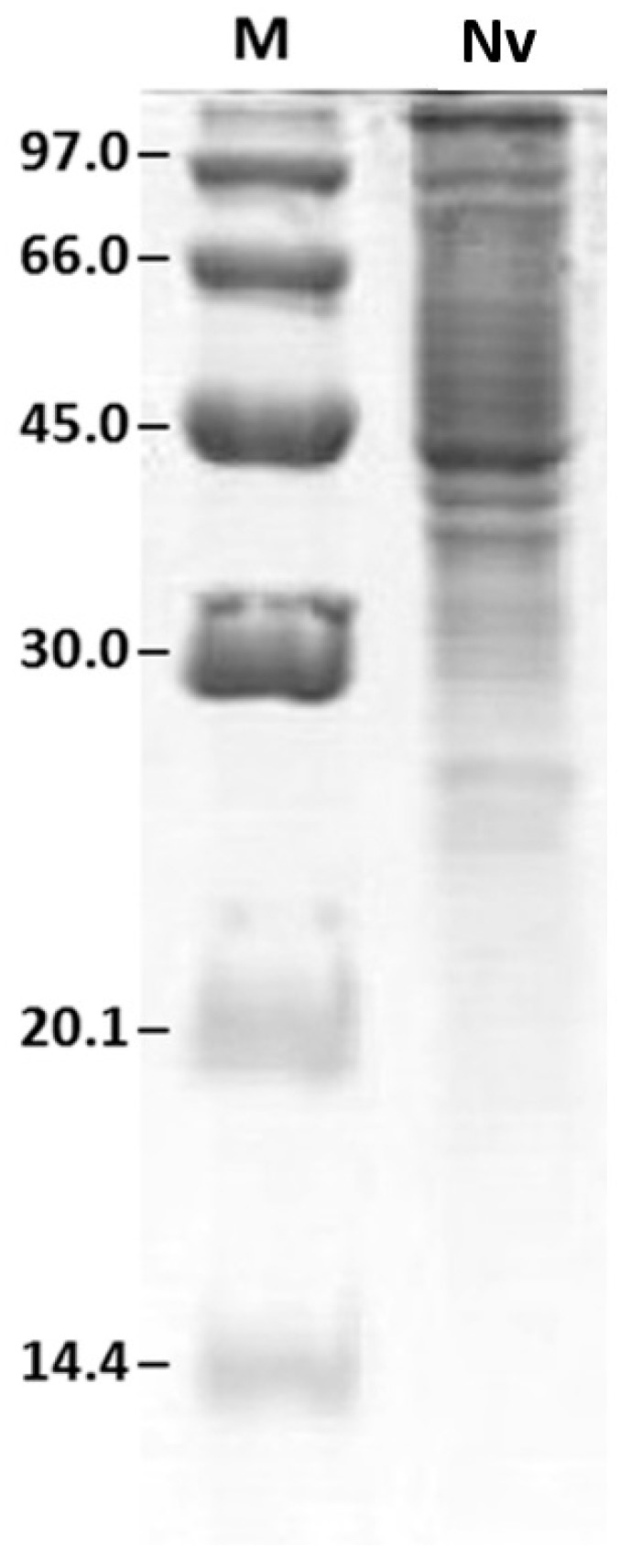
Protein profile of *Neoponera villosa* (*N. villosa*) venom by SDS-PAGE. M: molecular weight marker (values are displayed on the left, in kDa); Nv: venom of *N. villosa*.

Figure 1 shows the venom protein profile after SDS-PAGE.

The highest density of protein, shown by the largest number of bands, was observed between 20.1 and 97 kDa, revealing a predominance of higher molecular weight proteins.

### 2.3. Protein Profile Obtained by Two-Dimensional Electrophoresis (2D)

**Figure 2:**
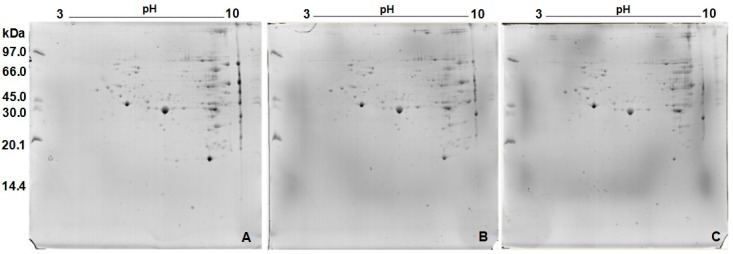
Two-dimensional gels of *Neoponera villosa* venom: (**A**) Replica 1 (Reference gel); (**B**) Replica 2; and (**C**) Replica 3.

**Figure 3:**
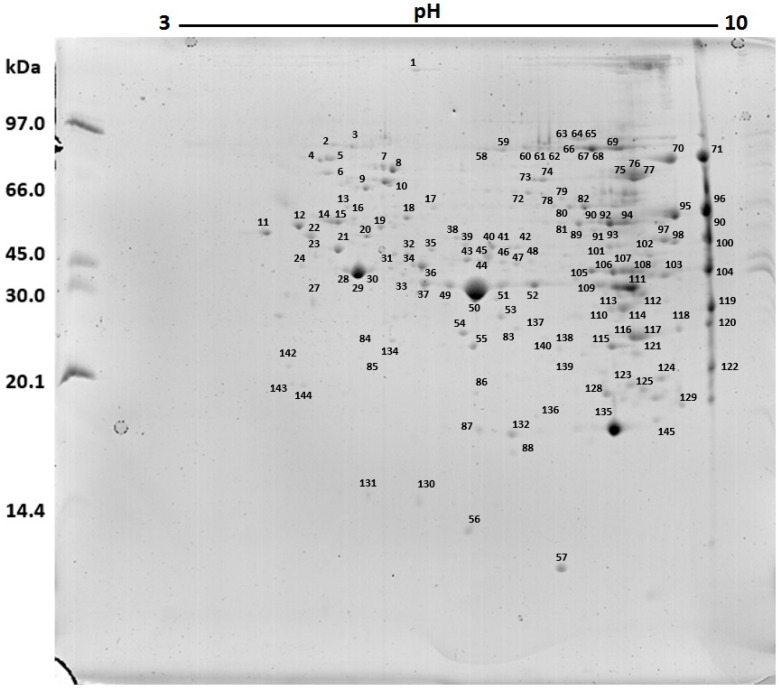
Spots corresponding to proteins identified in two-dimensional gel of *Neoponera villosa* venom.

The 2D gels (Figure 2) demonstrated a protein profile composed of 145 spots, from which 139 were identified (Figure 3 and Table 1).

### 2.4. Hemolytic Activity Induced by Neoponera villosa Venom

**Figure 4:**
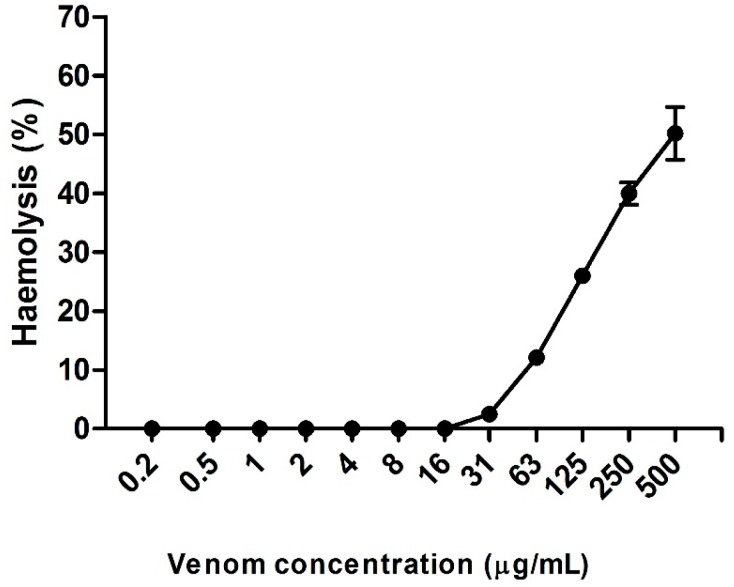
Percentage of hemolysis in different concentrations of *Neoponera villosa* venom.

Hemolysis was observed up to the fourth largest concentration of venom tested (63 µg/µL). Figure 4 presents the hemolysis rate of the venom in different concentrations. We can see that the hemolytic effect was dose-dependent, and was 50.6%, 40%, 26%, 12.1%, and 2.5% for the concentrations of 500, 250, 125, 63, and 31 µg/mL, respectively. Below these concentrations, it was not possible to observe hemolysis.

### 2.5. Induction of Cytokines in J774 Macrophages in the Presence of Neoponera villosa Venom

The presence of the venom in cultured murine macrophages was able to stimulate pro- and anti-inflammatory cytokines.

**Figure 5:**
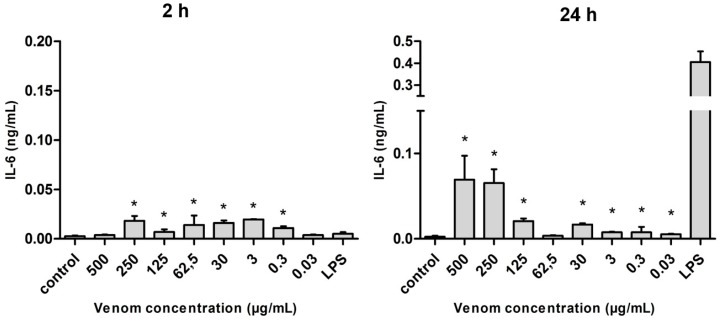
Amount of IL-6 (in ng/mL) released after the stimulus with *Neoponera villosa* venom. Values expressed in mean + standard deviation. Control: macrophages with supplemented culture medium alone. LPS: macrophages stimulated with 2 µg/mL LPS. * Denotes significantly different (*p* < 0.05).

The production of interleukin 6 (IL-6) by macrophages presented significantly higher levels in the presence of venom, especially after 24 h of stimulation. The difference in production was statistically significant at almost all the concentrations of venom used when compared with the negative control (with the exception of the highest and lowest concentrations studied in 2 h). However, within 24 h, the production levels of IL-6 were always lower than the value induced by lipopolysaccharide (LPS) (Figure 5).

**Figure 6:**
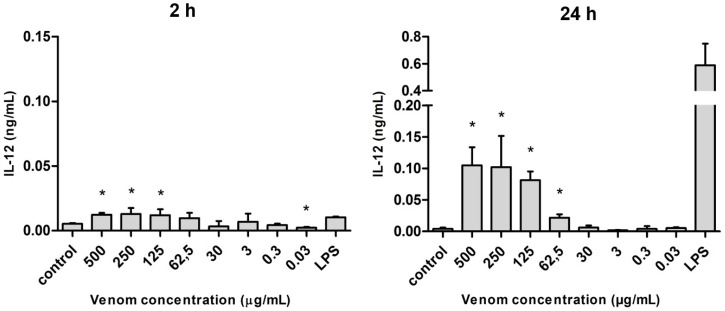
Amount of IL-12 (in ng/mL) released after the stimulus with *Neoponera villosa* venom Values expressed in mean + standard deviation. Control: macrophages with supplemented culture medium alone. LPS: macrophages stimulated with 2 µg/mL LPS. * Denotes significantly different (*p* < 0.05).

There was a larger release of interleukin 12 (IL-12) after 24 h of stimulation, which was statistically significant only at the highest concentrations of venom (500 to 62.5 µg/mL). Although the levels of IL-12 were discreet under 2-h stimulation, they were comparable to the amount found under LPS stimulus (Figure 6).

**Figure 7:**
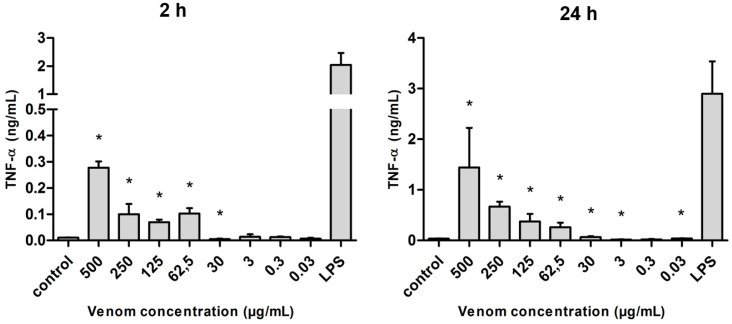
Amount of TNF-α (in ng/mL) released after the stimulus with *Neoponera villosa* venom. Values expressed in mean + standard deviation. Control: macrophages with supplemented culture medium alone. LPS: macrophages stimulated with 2 µg/mL LPS. * Denotes significantly different (*p* < 0.05).

Tumor necrosis factor α (TNF-α) presented the highest levels of production when compared with the other pro-inflammatory cytokines evaluated (IL-6 and IL-12). Regardless of the time of cultivation (2 or 24 h) and when compared with the basal condition, macrophages produced significantly greater amounts of TNF-α under stimulus of the *Neoponera villosa* venom. The production of this cytokine occurred in a dose-dependent way, as the concentration of the venom decreased, the amount of TNF-α detected also decreased. However, the highest levels of this cytokine under venom stimulus were still lower than those found under the LPS stimulus (Figure 7).

**Figure 8:**
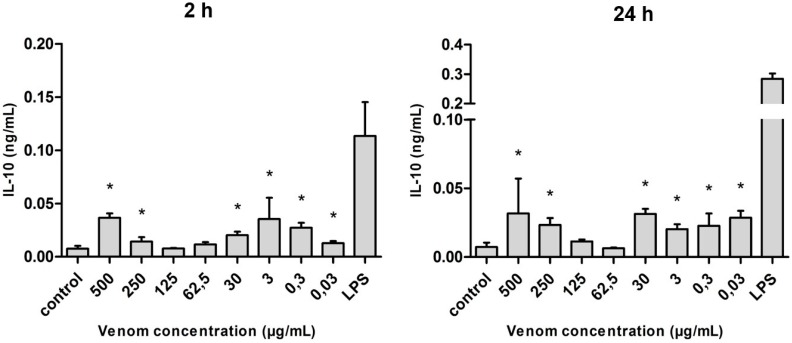
Amount of IL-10 (in ng/mL) released after the stimulus with *Neoponera villosa* venom. Values expressed in mean + standard deviation. Control: macrophages with supplemented culture medium alone. LPS: macrophages stimulated with 2 µg/mL LPS. * Denotes significantly different (*p* < 0.05).

Regarding interleukin 10 (IL-10), the venom was able to stimulate the production of this cytokine at all concentrations used, not demonstrating any relation to the dose of venom used on the cultures. The difference, statistically significant when compared with the control, was observed at all the concentrations used (with the exception of 125 and 62.5 µg/mL). In addition, the levels of IL-10 produced after 24 h of stimulation were slightly higher when compared with the 2-h stimulus, but they were not similar to those observed under LPS stimulus (Figure 8).

## 3. Discussion

The protein profile of the *N. villosa* venom showed predominance of proteins with higher molecular weight (between 20.1 and 97 kDa). Similarly, Lee *et al.* [25] worked with *Brachyponera chinensis* venom and observed higher protein density between 12 and 85 kDa. Silva *et al.* [31] analyzed *Odontomachus bauri* venom, and isolated proteins with molecular weight ranging from 18 to 160 kDa under nonreducing conditions, with more intense staining for bands above 29 kDa. Under reducing conditions, the electrophoretic profile changed, showing a wider range, from 24 to 160 kDa. The profile obtained by two-dimensional electrophoresis was composed of 145 spots. The distribution of these spots showed a higher density in neutral and alkaline areas and molecular weight above 20.1 kDa. To date, there are no data in the literature about protein profile of *N. villosa* venom; our results are the first ones. However, similar data were demonstrated in other species of the same genus. Costa Manso *et al.* [32] described, in the 2D profile of *N. goeldii* venom, higher density of spots between 10 and 66 kDa, and Lee *et al.* [25], studied *B. chinensis* venom and reported prevalence of proteins with pI in the neutral and alkaline zones. For the venom from another species of ant, *Solenopsis invicta*, all the proteins identified in 2D-SDS-PAGE were located above 14.4 kDa and most presented pI in the alkaline lane [33]. Such features are common to the venom of many hymenoptera as well as to ants, bees and wasps [34].

Regarding the 139 proteins identified in this study, it was observed that the venom houses proteins of several functional classes, from structural (e.g., actin), metabolic proteins (e.g., glyceraldehyde 3-phosphate-dehydrogenase), immunostimulatory molecules (e.g., heat shock proteins (HSPs)) to well described allergens (e.g., arginine kinase). Of these, the most biologically and biochemically relevant were: myosin (spot 1), endoplasmin (spot 2), HSPs (spots 5, 6, 10, 13), tubulin (spots 12, 14, 15, 16, 22, 41), α-*N*-acetylgalactosaminidase (spot 23), aspartyl protease (spot 27), actin (spots 28, 29, 30 and 31), arginine kinase (spots 33, 37, 38, 49, 50, 51, 52, 53, and 109), superoxide dismutase (spot 57), catalase (spot 95), hyaluronoglucosaminidase (spot 119), phospholipase A2 (spot 123), metalloproteinase (spot 132) and peroxiredoxin (spots 87 and 137).

In spots 2, 5, 6, 10, and 13, the following heat shock proteins (HSPs) or stress proteins were identified: HSP 90 (spots 2 and 5), HSP 70 (spots 6 and 10), and HSP 60 (spot 13). These proteins act as chaperones, facilitating the folding of other proteins, and as regulators of signaling, protecting the cell from the event of apoptosis [35]. In addition, the activation of the immune system mediated by HSPs is known. Preparations of purified HSPs, including HSP 60, HSP 70, and HSP 90, from various sources (wild and recombinant bacteria, several mammals, and humans) are potent activators of the innate immune system. These preparations induce the production of pro-inflammatory cytokines, such as TNF-α, IL-1, IL-6, and IL-12, in addition to nitric oxide by monocytes, macrophages and dendritic cells. [36,37]. The presence of such proteins in the venom may be related to the increased release of IL-12 and TNF-α by J774 1.6 macrophages, since, as demonstrated by Asea *et al.* [38], monocytes derived from human peripheral blood showed high levels of TNF-α, IL-1, and IL-6 release after the exposure to human HSP 70. Similarly, murine peritoneal macrophages and monocytes derived from human peripheral blood showed high expression of mRNA encoder of TNF-α, IL-1, IL-6, and GM-CSF after exposure to HSPs purified from several microorganisms such as *Escherichia coli*, *Mycobacterium tuberculosis*, and *Legionella pneumophila* [39,40].

In spot 23, α-*N*-acetylgalactosaminidase was identified, which is a hydrolase described for animals and bacteria [41]. It has already been described that this enzyme promotes immunosuppression through the inactivation of macrophages [42].

From spot 27, it was possible to identify an aspartyl protease, an enzyme with cathepsin D activity [43], capable of degrading a variety of substrates, including laminin and fibronectin, constituents of the extracellular matrix, thus contributing to the spread of other venom constituents through the body in which it is inoculated [44].

Several arginine kinase isoforms were identified (spots 33, 37, 38, 49, 50, 51, 52, 53, and 109). This protein has an enzymatic function and is responsible for the energetic metabolism in invertebrates. It is considered a pan-allergen, since it can trigger allergic responses through specific IgE linking [45]. It can be found in many allergenic sources, such as venoms, and has already been isolated from the venom of bees and wasps [35,46], as well as non-venomous animals used as food such as shrimp, octopus, and crab [47,48,49].

In spot 57, a superoxide dismutase (SOD) was identified as well as a catalase in spot 95. Both are responsible for the protecting the venom from the reactive oxygen species produced by the body, such as O_2_ and H_2_O_2_ [46]. In addition, a form of copper/zinc-dependent SOD was identified with the potential to trigger immune responses activation processes in the body where the venom is inoculated [50].

In spot 119, a hyaluronoglucosaminidase (or hyaluronidase) was identified, an enzyme present in several venoms and found in the venom of snakes, scorpions, bees, and wasps [51]. This enzyme acts on the cellular interstitial, downgrading it and is therefore, known as spreading factor [52]. It maximizes the infiltration of the venom as it digests part of the extracellular matrix, facilitating the action of other components [53].

A phospholipase A2 was identified from spot 123. This enzyme, widely distributed in several venoms, destroys phospholipids in biological membranes, leading to the formation of pores, cell lysis and tissue damage [33], inducing inflammation [54], and inhibiting the activity of macrophages [55]. The presence of the enzyme in the venom can be related to hemolytic activity and to inhibition of NO formation by macrophages.

Spot 132 has been identified as a metalloproteinase that is part of a family of enzymes involved in the occurrence of muscle necrosis, tissue damage, swelling and bleeding at the site of the sting, rare coagulopathies, among other related inflammatory reactions [56,57]. In addition, studies with venom from snakes have shown that the metalloproteinases, similar to hyaluronidases, act on the destruction of the extracellular matrix by interfering in cell adhesion, thus providing tissue disorganization [58].

In spots 87 and 137, forms of peroxiredoxin, belonging to a ubiquitous family of antioxidant proteins were identified [59]. Its presence in the venom is related to its own protection from oxidative stress. This function is also performed by glutathione *S*-transferase protein, found in spots 129 and 145 [33].

Structural proteins were identified in spots 1 (myosin), 12, 14, 15, 16, 22, 41 (tubulin) and 28, 29, 30, and 31 (actin). These proteins originate from muscle or tissues surrounding the venom reservoir. Studies by other authors suggest that the ant cells can rupture when the sting apparatus is removed from the insect with the aid of tweezers, which can cause the addition of these cell proteins to the venom proteins [46].

Although the remaining spots identified in the venom are classified exclusively as intracellular constituents (cytoplasmic or present inside organelles) and their presence in the venom is also related to the lesion of cells surrounding the venom reservoir during the mechanical removal of the glands [46], many of these proteins have been identified in proteomes of several venoms. In *Apis mellifera* venom, forms of malate dehydrogenase (spots 54 and 122), enolase (spots 35, 45, and 46) and apolipophorine (spot 77) were found [60]. In the venom of the endoparasitoid *Pteromalus puparum*, forms of vacuolar ATP synthase were found (spots 9, 19, and 21) and glyceraldehyde-3-phosphate dehydrogenase (spots 116, 117, and 118), among others [45]. Furthermore, Torres *et al.* [61] used transcriptomic approaches, and showed that more than 50% of the genes transcribed in the venom glands of the *Dinoponera quadriceps* ant were intracellular constituents.

Our study using a proteomic approach showed that *N. villosa* share many venom proteins with others ant species and even with other hymenopteran venoms. Similar proteomic tools were used by Touchard *et al.* [62] and they demonstrated that MALDI-TOF (Matrix-Assisted Laser Desorption/Ionization Time-Of-Flight) mass profiles of venom peptides from different species of *Neoponera*, *Pachycondyla* and *Odontomachus* permit taxonomic discrimination in closely related species. This chemotaxonomic tool is important in exploiting the enormous biodiversity of ants and other hymenopteran venoms as sources for novel drugs or biopesticides.

*Neoponera villosa* venom presented hemolytic activity which was dose-dependent, and the percentage of hemolysis was 50.6%, 40%, 26%, 12.1%, and 2.5% for concentrations of 500, 250, 125, 63, and 31 µg/mL, respectively. Below these concentrations, it was not possible to observe hemolysis. It has been shown that venoms from many organisms present hemolytic activity (in crude or fractionated form), among these organisms are: jellyfish, spiders, scorpions, and snakes [63,64,65,66].

Venoms from various species of hymenoptera also present hemolytic activity, such as *Pogonomyrmex barbatus* (*P. barbatus*) [18], *Paraponera clavata* (*P. clavata*) [21], *Myrmecia pilosula* (*M. pilosula*) [67], *Ectatomma tuberculatum* [68] ants, and wasps of the genus *Vespa* and *Polistes* [69]. The minimum concentrations of hemolysis vary and, within the same genus, there may be a great discrepancy. For example, it takes 500 U/mg dried venom from *P. barbatus* to provide 50% hemolysis [18], but it only takes 10 U/mg dried venom from *P. clavata* to obtain the same value of hemolysis [21]. Values close to those of the present study were found by Matuszek *et al.* [67], who took a concentration of 65 µg/mL venom from the *M. pilosula* ant to generate 12.5% hemolysis. The *Polistes lanio lanio* wasp requires a concentration equal to or greater than 50 µg/mL to obtain minimal hemolysis. Hemolysis may be the result of one or more mechanisms of action, and is usually linked to enzyme action. There may be direct hemolysis, in which one or more venom components act on the erythrocytic membrane, yielding pores and leading them to lysis, for example, there is the action of phospholipases that cleave the phospholipids of the membrane forming channels, where the intracellular content pours through [70] and proteins/peptides with surfactant activity [64]. Indirect hemolysis can be mediated by the action of complement, in which venom components, such as metalloproteinases, facilitate the action of cascade-signaling proteins [71].

Regarding *N. villosa* venom, a synergistic effect is suggested between the phospholipase and the metalloproteinase activities in the venom (spots 123 and 132). As these components are at low concentration in the venom, higher doses are required to obtain hemolysis (in this case, over 31 µg/mL).

The dosage of cytokines (IL-6, IL-10, IL-12, and TNF-α) released in the culture supernatant by macrophages exposed to the venom was performed by ELISA technique and, despite the significant increase in all pro-inflammatory cytokines (IL-6, IL-12, and TNF-α) in 2 and 24 h, there was also an increase of IL-10, a cytokine of anti-inflammatory and immunomodulatory character in both the periods evaluated. The induction pattern of pro-inflammatory cytokine has been observed for venom of several hymenoptera. Lam *et al.* [72] demonstrated that *A. mellifera* venom presents pro-inflammatory properties, inducing, *in vitro*, the release of cytokine IL-1 by macrophages through the activation of the p38 MAPK pathway. Tambourgi *et al.* [73] showed that, *in vivo*, the venom from the *Loxosceles intermedia* spider, is able to increase the IL-6 and TNF-α serum levels. Similar effect was observed by Malaque *et al.* [74], who stimulated primary culture human keratinocytes with venom from the *Loxosceles gaucho* spider and noted an increase in TNF-α. The IL-6 cytokine, as well as some chemokines, are also related to inflammation at the site of the sting induced by the venom from the same spider, as presented by Barbaro *et al.* [75]. The venom from the *Tityus serrulatus* scorpion generates local inflammation with high serum levels of IL-6 and TNF-α [12]. The same was observed, *in vitro*, when macrophages were exposed to the venom from the same species of scorpion and showed an increase in the release of IL-6 and TNF-α [76]. This pro-inflammatory pattern of the venom can be explained by the presence of some proteins known to induce macrophages to produce and release cytokines of this character, such as the heat shock proteins (HSPs), phospholipases, and metalloproteinases identified in this study. However, we also observed the production of IL-10, a cytokine that is anti-inflammatory and immunomodulatory in character able to inhibit the secretion of pro-inflammatory cytokines and regulate the differentiation and proliferation of some cells [77]. Some studies with venom also demonstrate increased IL-10 after stimulation. Fialho *et al.* [12] showed that in addition to the pro-inflammatory cytokines IL-6 and TNF-α, the venom from the scorpion *T. serrulatus* also induced an increase in serum IL-10. Andrade *et al.* [78] demonstrated that the stimulus with venom from the same scorpion raises the increased mRNA expression of both pro- (IL-1, IL-6, IFN-γ, and TNF-α) and anti-inflammatory (IL-10) cytokines. Ribeiro *et al.* [79] noted a similar fact and showed that after stimulating peripheral blood mononuclear cells with the venom from the snake *Crotalus durissus collilineatus*, there was an increase in the release of IL-10 and TNF-α as well. This fact could be related to the presence of components that stimulate both pro- and anti-inflammatory response in cells stimulated by the venom. Petricevich *et al.* [80] suggest that the production of IL-10 has a protective character in the process of poisoning by venom from snakes of the *Bothrops* genus, preventing the systemic shock condition.

## 4. Materials and Methods

### 4.1. Venom Extraction

The ants were collected while foraging on the campus of the State University of Santa Cruz (UESC) and on the Executive Committee of the Cocoa Crop (CEPLAC), in Ilhéus, Bahia, Brazil (14°47′20″S and 39°02′58″W). The insects were killed by freezing at −20 °C, identified in stereoscopic microscope and stored at −20 °C until use. We used 350 individuals (250 for protein characterization and 100 for biological activity tests). The crude venom, obtained by breaking the bag attached to the stinger after the ants’ dissection, was diluted in phosphate buffered saline (PBS) 100 mM pH 7.8.

### 4.2. Protein Extraction

To precipitate the proteins from the crude venom, the same amount of 20% (*v*/*v*) trichloroacetic acid (TCA) in acetone was added to each microtube containing venom. The microtube was packaged at −20 °C for 60 min and centrifuged at 16,100× *g* for 45 min to form the pellet. The supernatant was discarded. The pellet was washed with 500 µL cold acetone and then sonicated on ice for 21 s in cycles of 7 s with 70% range, and centrifuged for 10 min at 16,100× *g*, forming the pellet again. The supernatant was discarded and the pellet washed four times with acetone. The acetone was evaporated at room temperature and then rehydration buffer was added (7 M urea, 2 M thiourea, 2% CHAPS, and 0.002% bromophenol blue) to solubilize the proteins.

### 4.3. Protein Quantification

A 2D Quant Kit (GE Healthcare) was used to quantify proteins dissolved in the rehydration buffer, according to the manufacturer’s recommendations.

For the tests of biological activity (venom dissolved in PBS), dosage was used according to the Bradford protocol [30], using bovine serum albumin as standard.

### 4.4. Electrophoresis (SDS-PAGE)

For electrophoresis, 20 µg protein were subjected to SDS-PAGE (12.5% acrylamide). Aliquots of 7 µL of standard molecular weight (Amersham, Little Chalfont, UK) were also submitted to electrophoresis for comparison. After running, the gels were fixed (40% ethanol and 10% acetic acid) for 10 min, and then the solution was replaced by Coomassie Blue G-50 (Sigma Aldrich, St. Louis, MO, USA) dye, which was maintained for 12 h while stirring. Then, the dye was removed and the gels bleached with distilled water.

### 4.5. Two-Dimensional Electrophoresis (2D-SDS-PAGE)

Gel strips (Immobiline Dry Strip) of 13 cm pH 3-10 NL (GE Healthcare) were rehydrated for 12 h in rehydration buffer containing 350 µg protein. They were focused using a multiple-stage protocol (1 h at 500 V, 1 h at 1000 V and 6 h at 8000 V). Before the second dimension, each strip was balanced for 15 min in buffer containing 50 mM Tris-HCl, pH 8.8, 6 M urea, 30% glycerol, 2% SDS, 0.002% bromophenol blue, supplemented with 10 mg/mL DTT. After this stage, strips were balanced for 15 min in the buffer described above, but the DTT was replaced by 25 mg/mL of iodoacetamide. The second dimension electrophoresis was performed at 150 V using polyacrylamide gels (12.5%) in triplicate.

### 4.6. Protein Visualization and Image Analysis

Polyacrylamide gels were fixed (40% ethanol and 10% acetic acid) for 30 min, and then stained with Coomassie Blue G50 (Sigma) for five days while stirring. Subsequently, the gels were bleached with distilled water. Images of the gels were scanned and analyzed by ImageMaster 2D Platinum 7.0 (GE Healthcare) software to assess the number of spots, and characterize the values of pI and molecular weight.

### 4.7. Spot Excision and Triptych Digestion

The spots were excised from the gels and arranged separately in microtubes. Later, they were added to the gel fragments 200 µL NH_4_HCO_3_ (ammonium bicarbonate), 25 mM, pH 8.0 in 50% acetonitrile to discolor and remove SDS. This solution was kept in the microtubes for 24 h. After this period, the spots were washed with 200 µL ultrapure water, 100 µL acetonitrile were added to dehydrate the gel, and the fragments were incubated for 5 min at room temperature. The excess acetonitrile was removed, and the microtubes were vacuum dried for 20 min. Then, the proteins were partially digested with trypsin (5 µL at the concentration of 20 ng/µL) for 10 min at 4 °C. Later, 200 µL NH_4_HCO_3_, 25 mM were added, followed by incubation at 37 °C for 24 h to complete the digestion of the peptides. To extract the peptides, the remaining solution was transferred to a differente and clean microtube, and 50 µL 50% acetonitrile were added to the first microtube containing 0.1% formic acid. These microtubes containing a mixture of acetonitrile, formic acid, and residual peptides were agitated slightly for 30 min. The solution was pipetted to the new microtubes, and this process was repeated. In the end, the microtubes containing fully digested peptides were vacuum dried to the volume of 5–10 µL.

### 4.8. Peptide Sequencing and Protein Identification

The peptides obtained as described in the previous item were separately applied in the nanoAcquity UPLC (Waters, Milford, MA, USA) in two C18 columns (“trapping” of 5 µm, 180 µm × 20 mm; and the second of 1.7 µm, 100 µm × 100 mm), under a stream of 0.6 µL/min in a 50-min run. The peptides were separated according to acetonitrile gradient grade, transferred to the Mass Spectrometer (Micromass Q-TOFmicro, Waters) and ionized in a capillary under 3000 V. They were fragmented in the positive mode with selection of minimum relative intensity of 10 counts, and the three most intense ions were analyzed per every 1 s sweep, with collision energy ranging from 20 to 95 V according to the mass/charge (*m*/*z*) of peptides. The spectra obtained in each run were analyzed by MASCOT Server Online (Matrix Science: http://www.matrixscience.com/), and compared with the NCBI database. On hydrolysis, by trypsin, the possible loss of a cleavage site was considered. The tolerance of the peptide masses was ±0.3 Da, and the tolerance of the fragment masses was ±0.1 Da.

### 4.9. Hemolytic Activity

To perform the test, a 96-well microplate with a U-shaped bottom was used. The initial venom concentration was 500 µg/mL and was performed a serial dilution to the concentration of 0.2 µg/mL venom. Then, 100 µL red blood cell suspension was added to 1% (*v*/*v*). For the reference value of zero, 100 µL saline solution and 100 µL red blood cell suspension were added. For the reference value of 100% hemolysis, 100 µL saline solution containing 1% (*v*/*v*) Triton X-100 (Sigma) and 100 µL red blood cell suspension were added. The plates were incubated at 25 °C for 120 min. Then, the microplates were centrifuged for 5 min at 1440× *g*. The supernatant of each well was collected and transferred to the wells of a new plate with a flat bottom. The rate of hemolysis was taken by the absorbance reading at 540 nm and the hemolysis was calculated by the following equation:

$$Haemolysis\;\left(\%\right)=\;\frac{Adilution-Aneg.control}{Aposit.control-Aneg.control}\times 100$$

### 4.10. Murine Macrophages Culture and Stimulus with Neoponera villosa Venom

J774 1.6 murine macrophages (Rio de Janeiro Cell Bank—BCRJ, access No. 0273) were cultured for 24 h until they reached the confluence amid DMEM (Gibco, Waltham, MA, USA) supplemented with 10% fetal bovine serum (FBS) (Gibco) and 1% antibiotics (penicillin and streptomycin) (Gibco), at 37 °C, 5% CO_2_ in the concentration of 1 × 10^6^ cells/mL, and the viability was monitored with trypan blue dye at each bouncing.

For the tests with venom stimulation, the macrophages were cultured for 48 h at the concentration of 1 × 10^6^ cells/mL in 24-well plates containing DMEM, 10% FBS and 1% antibiotics as well as different concentrations of venom (ranging from 500 to 0.03 μg/mL). As positive stimulus control the cells were grown in the presence 2 μg/mL of bacterial lipopolysaccharide (LPS) (Sigma) and as negative control (basal condition) only with the culture supplemented with FBS and antibiotic.

### 4.11. Cytokines Doses

Five hundred milliliter aliquots from culture supernatants described in item 4.10 were removed, in periods of 2 and 24 h after the stimulus. Cytokines IL-6, IL-10, IL-12, and TNF-α were evaluated by ELISA kits (Peprotech, Ribeirão Preto, São Paulo, Brazil), according to the manufacturer’s recommendations.

The statistical analysis of cytokine concentration was carried out via the GraphPad Prism 5 software, using the Mann–Whitney non-parametric test, considering *p* < 0.05 and confidence interval (CI) of 95%.

## 5. Conclusions

The venom of *Neoponera villosa* has a complex protein composition, which consists of 145 proteins in the neutral and alkaline lanes of pH and above 20.1 kDa. In addition, *N. villosa* venom presents hemolytic activity for concentrations exceeding 31 µg/µL and this fact is related to the presence of phospholipase and metalloproteinase enzymes in the venom (spots 123 and 132, respectively). *N. villosa* venom induces an increase in the release of pro-inflammatory cytokines (IL-6, IL-8, and TNF-α), and this increase is related to the presence of the following proteins in the venom: HSP 90 (spots 2 and 5), HSP 70 (spots 6 and 10), and HSP 60 (spot 13), since it is known that HSPs are potent activators of the innate immune system, inducing the release of pro-inflammatory cytokines. In addition to the pro-inflammatory cytokines, there was slight increase in IL-10 production by macrophages, and this fact could be related to the presence of components that stimulate both a pre- and anti-inflammatory response in the venom or with a cellular compensatory mechanism aimed at preventing damages caused by the high concentration of pro-inflammatory cytokines. These results allow a better understanding of the composition and complexity of *N. villosa* venom in the human body, as well as the venom’s possible mechanisms of action after the bite.

## Figures and Tables

**Table 1:** Identified proteins in *Neoponera villosa* venom. MW: Molecular weight.

Spot	Protein/Source	Accession Code	*M*w (Da)	pI	Coverage/Score
1	Myosin heavy chain, muscle-like isoform 2/*Bombus terrestris* (bee)	gi|340718034	251,060	5.58	15%/1509
2	Endoplasmin/*Harpegnathos saltator* (ant)	gi|307192149	90,928	4.96	22%/907
3	Transitional endoplasmic reticulum ATPase TER94/*Camponotus floridanus* (ant)	gi|307174120	89,511	5.14	37%/1213
4	Hemocyte protein-glutamine gamma-glutamyltransferase/*Harpegnathos saltator* (ant)	gi|307215415	79,590	4.89	36%/1291
5	Heat shock protein HSP 90-α/*Camponotus floridanus* (ant)	gi|307186382	83,640	4.98	40%/1410
6	Heat shock 70 kDa protein cognate 3/*Harpegnathos saltator* (ant)	gi|307210158	72,646	5.02	44%/1709
7	Transferrin/*Harpegnathos saltator* (ant)	gi|307215135	82,014	5.72	15%/617
8	Transferrin/*Harpegnathos saltator* (ant)	gi|307215135	82,014	5.72	17%/718
9	Vacuolar ATP synthase catalytic subunit A/*Harpegnathos saltator* (ant)	gi|307196686	68,591	5.25	60%/2338
10	Heat shock protein cognate 4 (70 kDa)/*Apis mellifera* (bee)	gi|229892210	71,383	5.43	50%/2204
11	Calreticulin/*Harpegnathos saltator* (ant)	gi|307191961	47,000	4.42	38%/810
12	Tubulin β-1 chain/*Harpegnathos saltator* (ant)	gi|307201205	49,010	4.67	40%/818
13	60 kDa heat shock protein, mitochondrial/*Harpegnathos saltator* (ant)	gi|307199045	64,756	5.32	35%/1012
14	Tubulin α-1 chain-like/*Nasonia vitripennis* (wasp)	gi|156548149	50,647	5.01	56%/1598
15	Tubulin α-1 chain/*Harpegnathos saltator* (ant)	gi|307208702	52,080	5.00	51%/1453
16	Putative α-tubulin/*Maconellicoccus hirsutus* (mealybug)	gi|121544009	50,633	5.01	25%/451
17	Phosphoglucomutase/*Acromyrmex echinatior* (ant)	gi|332024861	66,241	6.20	12%/287
18	Protein disulfide-isomerase A3/*Harpegnathos saltator* (ant)	gi|307194521	56,272	5.53	11%/260
19	Vacuolar ATP synthase subunit B/*Camponotus floridanus* (ant)	gi|307174076	55,263	5.37	58%/1628
20	Eukaryotic initiation factor 4A-II/*Camponotus floridanus* (ant)	gi|307189936	48,514	5.30	47%/1002
21	ATP synthase subunit β, mitochondrial/*Harpegnathos saltator* (ant)	gi|307195440	55,223	5.32	64%/2075
22	Tubulin β-1 chain/*Apis mellifera* (bee)	gi|48095525	50,599	4.75	65%/1193
23	α-*N*-acetylgalactosaminidase/*Harpegnathos saltator* (ant)	gi|307213390	161,528	5.99	6%/368
24	cAMP-dependent protein kinase type I regulatory subunit isoform 1/*Apis mellifera* (bee)	gi|48106841	41,923	4.86	34%/611
27	Lysosomal aspartic protease/*Acromyrmex echinatior* (ant)	gi|332024025	42,132	5.34	13%/254
28	Actin-5, muscle-specific/*Harpegnathos saltator* (ant)	gi|307197034	42,098	5.30	68%/1145
29	Actin, muscle/*Camponotus floridanus* (ant)	gi|307166491	42,071	5.29	71%/1252
30	Actin, muscle/*Camponotus floridanus* (ant)	gi|307166491	42,071	5.29	62%/955
31	Actin/*Aedes aegypti* (mosquito)	gi|94468486	42,164	5.30	40%/484
32	Rab GDP dissociation inhibitor β/*Camponotus floridanus* (ant)	gi|307172388	50,104	5.51	36%/757
33	Arginine kinase/*Acromyrmex echinatior* (ant)	gi|332018357	40,032	5.86	18%/313
34	Mitochondrial-processing peptidase subunit β/*Harpegnathos saltator* (ant)	gi|307207091	53,918	5.77	28%/572
35	Enolase/*Harpegnathos saltator* (ant)	gi|307211488	47,379	5.79	40%/650
36	Succinyl-CoA ligase (ADP-forming) subunit β, mitochondrial-like/*Bombus terrestris* (bee)	gi|340726331	49,012	6.63	31%/861
37	Arginine kinase/*Harpegnathos saltator* (ant)	gi|307197996	39,996	5.75	45%/1201
38	RecName: Full = Arginine kinase; Short = AK/*Homarus gammarus* (lobster)	gi|585342	40,300	6.05	4%/88
39	Retinal dehydrogenase 1/*Acromyrmex echinatior* (ant)	gi|332024132	53,459	6.14	9%/283
40	Retinal dehydrogenase 1/*Acromyrmex echinatior* (ant)	gi|332024132	53,459	6.14	9%/333
41	α-Tubulin at 84B/*Drosophila melanogaster* (fly)	gi|17136564	50,561	5.00	25%/402
42	Retinal dehydrogenase 1/*Acromyrmex echinatior* (ant)	gi|332024132	53,459	6.14	9%/333
43	Aminoacylase-1/*Harpegnathos saltator* (ant)	gi|307206409	45,783	5.66	16%/319
44	Adenosylhomocysteinase/*Harpegnathos saltator* (ant)	gi|307206413	48,440	5.88	30%/737
45	Enolase/*Harpegnathos saltator* (ant)	gi|307211488	47,379	5.79	44%/778
46	Enolase/*Harpegnathos saltator* (ant)	gi|307211488	47,379	5.79	16%/307
47	Phosphoglycerate kinase/*Camponotus floridanus* (ant)	gi|307177429	45,039	6.16	35%/1031
48	Phosphoglycerate kinase/*Camponotus floridanus* (ant)	gi|307177429	45,039	6.16	23%/416
49	Arginine kinase/*Harpegnathos saltator* (ant)	gi|307197996	39,996	5.75	47%/1381
50	Arginine kinase/*Harpegnathos saltator* (ant)	gi|307197996	39,996	5.75	41%/1045
51	Arginine kinase/*Harpegnathos saltator* (ant)	gi|307197996	39,996	5.75	45%/637
52	Arginine kinase/*Harpegnathos saltator* (ant)	gi|307197996	39,996	5.75	39%/652
53	Arginine kinase/*Acromyrmex echinatior* (ant)	gi|332018357	40,032	5.86	10%/211
54	Malate dehydrogenase, cytoplasmic/*Camponotus floridanus* (ant)	gi|307166391	39,169	7.03	20%/533
55	Glycerol-3-phosphate dehydrogenase (NAD+), cytoplasmic/*Acromyrmex echinatior* (ant)	gi|332024225	37,294	7.15	44%/1106
56	Glycerol-3-phosphate dehydrogenase (NAD+), cytoplasmic/*Acromyrmex echinatior* (ant)	gi|332024225	37,294	7.15	25%/290
57	Superoxide dismutase (Cu-Zn)/*Harpegnathos saltator* (ant)	gi|307204104	14,008	6.18	43%/213
58	Glycogen phosphorylase/*Harpegnathos saltator* (ant)	gi|307199215	121,697	6.22	17%/837
59	Glycogen phosphorylase/*Harpegnathos saltator* (ant)	gi|307199215	121,697	6.22	20%/1060
60	Cytoplasmic aconitate hydratase/*Harpegnathos saltator* (ant)	gi|307196718	98,222	6.11	11%/319
61	10-formyltetrahydrofolate dehydrogenase/*Acromyrmex echinatior* (ant)	gi|332029989	99,956	5.88	4%/139
62	Elongation factor 2/*Camponotus floridanus* (ant)	gi|307170298	94,303	6.11	16%/496
63	2-oxoglutarate dehydrogenase E1 component, mitochondrial/*Acromyrmex echinatior* (ant)	gi|332017156	121,347	6.78	13%/541
64	2-oxoglutarate dehydrogenase E1 component, mitochondrial/*Acromyrmex echinatior* (ant)	gi|332017156	121,347	6.78	25%/1252
65	2-oxoglutarate dehydrogenase E1 component, mitochondrial/*Acromyrmex echinatior* (ant)	gi|332017156	121,347	6.78	26%/1262
66	Filamin-C/*Harpegnathos saltator* (ant)	gi|307210403	252,199	6.12	14%/1364
67	Filamin-C/*Harpegnathos saltator* (ant)	gi|307210403	252,199	6.12	17%/1704
68	Filamin-C/*Harpegnathos saltator* (ant)	gi|307210403	252,199	6.12	18%/2261
69	Filamin-C/*Harpegnathos saltator* (ant)	gi|307210403	252,199	6.12	18%/1008
70	Aconitate hydratase, mitochondrial/*Harpegnathos saltator* (ant)	gi|307201595	85028	8.36	25%/839
71	Aconitate hydratase, mitochondrial/*Harpegnathos saltator* (ant)	gi|307201595	85028	8.36	36%/1333
72	Succinate dehydrogenase (ubiquinone) flavoprotein subunit, mitochondrial/*Acromyrmex echinatior* (ant)	gi|332019677	73506	6.75	27%/886
73	Putative actin-interacting protein 1/*Harpegnathos saltator* (ant)	gi|307210939	69910	5.69	36%/1180
74	Putative actin-interacting protein 1/*Harpegnathos saltator* (ant)	gi|307210939	69910	5.69	29%/708
75	Glycerol-3-phosphate dehydrogenase, mitochondrial/*Harpegnathos saltator* (ant)	gi|307212068	81972	6.51	16%/530
76	Glycerol-3-phosphate dehydrogenase, mitochondrial/*Harpegnathos saltator* (ant)	gi|307212068	81972	6.51	13%/292
77	Apolipophorins/*Harpegnathos saltator* (ant)	gi|307201472	382,345	6.47	1%/400
78	NADP-dependent malic enzyme/*Harpegnathos saltator* (ant)	gi|307205633	66,782	7.19	29%/763
79	NADP-dependent malic enzyme/*Harpegnathos saltator* (ant)	gi|307205633	66,782	7.19	13%/207
80	NADP-dependent malic enzyme/*Harpegnathos saltator* (ant)	gi|307205633	66,782	7.19	49%/1238
81	Dihydrolipoyl dehydrogenase, mitochondrial/*Harpegnathos saltator* (ant)	gi|307209020	54,499	6.87	7%/117
82	NADP-dependent malic enzyme/*Harpegnathos saltator* (ant)	gi|307205633	66,782	7.19	54%/1447
83	NADP-dependent malic enzyme/*Harpegnathos saltator* (ant)	gi|307205633	66,782	7.19	12%/195
84	Transaldolase/*Harpegnathos saltator* (ant)	gi|307215256	37,393	5.27	7%/102
85	Pyruvate dehydrogenase E1 component subunit β, mitochondrial/*Harpegnathos saltator* (ant)	gi|307195718	39,486	5.87	34%/475
86	Proteasome subunit β type-7/*Harpegnathos saltator* (ant)	gi|307192825	30,622	7.00	28%/250
87	Peroxiredoxin 1/*Camponotus floridanus* (ant)	gi|307175821	21,887	6.30	32%/423
88	Proteasome subunit β type-1/*Harpegnathos saltator* (ant)	gi|307214019	26,343	6.81	20%/108
89	Dihydrolipoyl dehydrogenase, mitochondrial/*Harpegnathos saltator* (ant)	gi|307209020	54,499	6.87	23%/491
90	Glutamate dehydrogenase, mitochondrial/*Harpegnathos saltator* (ant)	gi|307195623	62,095	8.40	27%/549
91	Selenium-binding protein 1-A/*Acromyrmex echinatior* (ant)	gi|332021867	52,719	6.99	8%/233
92	Glutamate dehydrogenase, mitochondrial/*Harpegnathos saltator* (ant)	gi|307195623	62,095	8.40	52%/1140
93	Glutamate dehydrogenase, mitochondrial/*Harpegnathos saltator* (ant)	gi|307195623	62,095	8.40	53%/1166
94	Glutamate dehydrogenase, mitochondrial/*Harpegnathos saltator* (ant)	gi|307195623	62,095	8.40	52%/1356
95	Catalase/*Harpegnathos saltator* (ant)	gi|307197480	58,234	8.58	23%/170
96	Elongation factor-1 α, partial/*Hoplitis ochraceicornis* (bee)	gi|426322152	40,701	8.58	41%/226
97	Alanine aminotransferase 2/*Harpegnathos saltator* (ant)	gi|307214462	61,520	9.26	24%/665
98	ATP synthase subunit α, mitochondrial/*Harpegnathos saltator* (ant)	gi|307208992	59,687	9.12	41%/1271
99	Elongation factor 1-α/*Harpegnathos saltator* (ant)	gi|307196337	50,620	9.13	43%/350
100	ATP synthase subunit α, mitochondrial/*Harpegnathos saltator* (ant)	gi|307208992	59,687	9.12	32%/364
101	4-hydroxybutyrate coenzyme A transferase/*Harpegnathos saltator* (ant)	gi|307203837	53,231	7.69	36%/769
102	Probable fumarate hydratase, mitochondrial/*Harpegnathos saltator* (ant)	gi|307202128	47,153	8.14	24%/478
103	Probable citrate synthase 1, mitochondrial/*Harpegnathos saltator* (ant)	gi|307202019	49,316	8.94	34%/788
104	Probable citrate synthase 1, mitochondrial/*Harpegnathos saltator* (ant)	gi|307202019	49,316	8.94	37%/471
105	Isocitrate dehydrogenase (NADP) cytoplasmic-like isoform 1/*Bombus terrestris* (bee)	gi|340721268	46,518	8.00	9%/146
106	Isocitrate dehydrogenase (NADP) cytoplasmic/*Harpegnathos saltator* (ant)	gi|307210166	50,629	8.20	44%/952
107	Isocitrate dehydrogenase (NADP) cytoplasmic/*Harpegnathos saltator* (ant)	gi|307210166	50,629	8.20	41%/611
108	Isocitrate dehydrogenase (NADP) cytoplasmic/*Harpegnathos saltator* (ant)	gi|307210166	50,629	8.20	19%/422
109	Arginine kinase /*Harpegnathos saltator* (ant)	gi|307197996	39,996	5.75	46%/1213
110	Fructose-bisphosphate aldolase-like/*Megachile rotundata* (bee)	gi|383861513	40,009	7.00	18%/470
111	Four and a half LIM domains protein 2/*Acromyrmex echinatior* (ant)	gi|332021158	28,335	8.46	52%/758
112	Isocitrate dehydrogenase (NAD) subunit β, mitochondrial/*Harpegnathos saltator* (ant)	gi|307210578	41,512	8.81	31%/530
113	Sorbitol dehydrogenase/*Harpegnathos saltator* (ant)	gi|307204829	38,256	7.56	27%/488
114	Fructose-bisphosphate aldolase-like/*Megachile rotundata* (bee)	gi|383861513	40,009	7.00	33%/635
115	Aldose reductase/*Camponotus floridanus* (ant)	gi|307181859	35,897	6.11	17%/212
116	Glyceraldehyde-3-phosphate dehydrogenase 2/*Harpegnathos saltator* (ant)	gi|307198667	35,769	7.64	45%/898
117	Glyceraldehyde-3-phosphate dehydrogenase 2/*Harpegnathos saltator* (ant)	gi|307198667	35,769	7.64	45%/848
118	Glyceraldehyde-3-phosphate dehydrogenase 2/*Harpegnathos saltator* (ant)	gi|307198667	35,769	7.64	42%/603
119	Hyaluronoglucosaminidase/*Camponotus floridanus* (ant)	gi|307180582	40,178	8.45	10%/216
120	Succinyl-CoA ligase (GDP-forming) subunit α, mitochondrial/*Harpegnathos saltator* (ant)	gi|307203732	34,525	8.70	23%/476
121	Guanine nucleotide-binding protein subunit β-like protein/*Harpegnathos saltator* (ant)	gi|307215409	36,333	7.63	68%/913
122	Malate dehydrogenase, mitochondrial/*Harpegnathos saltator* (ant)	gi|307214026	36,178	9.36	46%/670
123	Phospholipase A2 isozymes PA3A/PA3B/PA5/*Apis mellifera* (bee)	gi|328778177	26,379	7.46	6%/103
124	Proteasome subunit α type-7-1/*Harpegnathos saltator* (ant)	gi|307201582	28,118	6.97	32%/385
125	Dehydrogenase/reductase SDR family member 11/*Harpegnathos saltator* (ant)	gi|307212910	17,798	9.61	9%/102
128	Phosphoglycerate mutase 2-like/*Bombus terrestris* (bee)	gi|340726229	35,071	9.16	36%/733
129	Glutathione S-transferase sigma 3/*Locusta migratoria* (locust)	gi|329564873	23,498	7.63	8%/94
130	Adenylate kinase isoenzyme F38B2.4/*Harpegnathos saltator* (ant)	gi|307204436	21,291	5.42	35%/525
131	Protein DJ-1/*Camponotus floridanus* (ant)	gi|307174129	20,023	5.93	7%/110
132	RecName: Full = Snake venom metalloproteinase atroxlysin-1; Short = SVMP; AltName: Full = Atroxlysin-I/*Bothrops atrox* (snake)	gi|353526296	23,302	5.85	12%/135
134	Transaldolase/*Harpegnathos saltator* (ant)	gi|307215256	37,393	5.27	7%/168
135	Peroxiredoxin-6/*Harpegnathos saltator* (ant)	gi|307213913	25,248	6.65	18%/124
136	Triosephosphate isomerase/*Harpegnathos saltator* (ant)	gi|307199049	26,956	6.00	49%/695
137	Eukaryotic translation initiation factor 3 subunit I/*Acromyrmex echinatior* (ant)	gi|332024413	36,384	6.00	25%/210
138	Glycine *N*-methyltransferase/*Harpegnathos saltator* (ant)	gi|307192516	33,820	6.26	19%/259
139	Fumarylacetoacetate hydrolase domain-containing protein 2A/*Harpegnathos saltator* (ant)	gi|307203489	37,311	8.54	8%/109
140	Ribose-phosphate pyrophosphokinase 1/*Harpegnathos saltator* (ant)	gi|307207320	37,856	6.26	23%/320
142	14-3-3 protein epsilon-like/*Nasonia vitripennis* (wasp)	gi|156553657	29,323	4.72	4%/123
143	Putative 14-3-3 protein/*Maconellicoccus hirsutus* (mealybug)	gi|121543925	28,171	4.80	9%/83
144	14-3-3 protein ζ/*Camponotus floridanus* (ant)	gi|307186410	35,115	5.01	11%/149
145	Glutathione S-transferase 1, isoform C/*Harpegnathos saltator* (ant)	gi|307196173	24,899	7.71	18%/175

## Abbreviations

2D	Two-dimensional
BCRJ	Rio de Janeiro Cell Bank
CEPLAC	Executive Committee of the Cocoa Crop
CHAPS	3-((3-Cholamidopropyl)dimethylammonio)-1-propanesulfonate
CI	Confidence interval
DMEM	Dulbecco’s Modified Eagle’s Medium
DTT	Dithiothreitol
ELISA	Enzyme linked immuno sorbent assay
FBS	Fetal bovine serum
HSP	Heat shock proteins
IL	Interleukin
LPS	Lipopolysaccharide
MALDI-TOF	Matrix-Assisted Laser Desorption/Ionization Time-Of-Flight
MAPK	Mitogen-activated protein kinase
MW	Molecular weight
NCBI	National Center for Biotechnology Information
NO	Nitric oxide
PAGE	Polyacrylamide gel electrophoresis
PBS	Phosphate buffered saline
pI	Isoelectric point
SOD	Superoxide dismutase
SDS	Sodium dodecyl sulfate
TCA	Trichloroacetic acid
TNF	Tumor necrosis factor
UESC	State University of Santa Cruz
UPLC	Ultra performance liquid chromatography
